# A Life Prediction Model of Multilayered PTH Based on Fatigue Mechanism

**DOI:** 10.3390/ma10040382

**Published:** 2017-04-04

**Authors:** Yaqiu Li, Weiwei Hu, Yufeng Sun, Zili Wang, Ali Mosleh

**Affiliations:** 1 Reliability and System Engineering School, Beihang University, Haidian District, Beijing 100191, China; ermao13@163.com (Y.L.); syf@buaa.edu.cn (Y.S.); wzl@buaa.edu.cn (Z.W.); 2 B. John Garrick Institute for the Risk Sciences, University of California, Los Angeles, CA 90095, USA; mosleh@ucla.edu

**Keywords:** PTH, multilayer, stress-strain model, fatigue life prediction, FEA, thermal cycling test

## Abstract

Plated through hole (PTH) plays a critical role in printed circuit board (PCB) reliability. Thermal fatigue deformation of the PTH material is regarded as the primary factor affecting the lifetime of electrical devices. Numerous research efforts have focused on the failure mechanism model of PTH. However, most of the existing models were based on the one-dimensional structure hypothesis without taking the multilayered structure and external pad into consideration. In this paper, the constitutive relation of multilayered PTH is developed to establish the stress equation, and finite element analysis (FEA) is performed to locate the maximum stress and simulate the influence of the material properties. Finally, thermal cycle tests are conducted to verify the accuracy of the life prediction results. This model could be used in fatigue failure portable diagnosis and for life prediction of multilayered PCB.

## 1. Introduction

With the miniaturization of printed circuit board scale and the increase in package density, electronic interconnection malfunctions occur more frequently. Plated through hole (PTH) is considered to be one of the most critical factors that causes interconnection failure. Typically, the primary reason of PTH failure is the difference between the CTE (coefficient of thermal expansion) of the substrate and plating material, which generates a cyclic tension-compression force inside the plating layer under thermal loads according to the constitutive relation, then produces thermal fatigue deformation, and finally leads to the PTH and even the whole electrical device failure. As such, the physics factors (material, geometry, etc.) of failure play a vital role in PTH reliability. To investigate the failure mechanism of PTH, many studies proposed different models over the years. The first was a kind of one-dimensional [1] rod model, which was proposed to estimate the stress-strain response of plated through hole [2], based on which the residual strain component after thermal cycle load was predicted. Reference [3] gave a stress-strain analytical equation in the elastic-plastic range, and proposed two major experimental measurements of structural failure mechanisms. However, this model did not satisfy the free boundary condition of plating surface and the continuous displacement rule between plating wall and substrate bonding. Xie [4] improved this model by taking the shear stress of the copper-resin surface into consideration, where the improved model analyzed the PTH’s stress-strain distribution in the axial direction and offered an analytical relationship between fatigue life and influencing factors (including material and geometry properties). Subsequent publications by others proposed more assumption on structure to optimize the model. Reference [5,6] set the pad structure as beam structure and assumed that axial strain and stress in the board thickness direction were consistent. They then analyzed the effect of different parameters on PTH reliability during the thermal cycle. The finite element method [7,8,9] and Bayesian approaches [10,11] have been investigated with regard to the relationship between fatigue life and degradation influenced factors of electronic devices. Remarkably, many researches on fatigue lifetime estimation are concentrated on combining the physics-based models together with monitored or test data under a probabilistic framework [12,13,14], which can illuminate and qualify the uncertainty of fatigue failure mechanism effectively under synthetic contributions (such as environment, load, structure, material, manufacture defection, and human factors). Knadle [15] used an environmental scanning electron microscope (SEM) to photograph and even video tape the opening and closing of a PTH crack during a reflow cycle, using a Coffin-Manson model for life prediction. Sun [16] conducted a sensitivity analysis method for fatigue parameters of PTH during a thermal cycle by considering the uncertainty sources of parameter fluctuation. Pan [17] used the Bayesian approach to analyze the influence of PTH low-cycle fatigue failure in printed wiring boards. Chen [18] investigated the effects of new ingredient l in copper plating on the thermal reliability of PTH.

However, the fatigue model of PTH involved in previous research seems not to be so widely applicable now because of the inadequacy of one-dimensional rod hypothesis. Therefore, the stress-stain model of PTH in a multilayered structure is investigated both numerically and experimentally in this paper. The theoretical formula of the constitutive relation of PTH in a multilayered structure is developed and finite element stimulation and thermal cycling tests are performed to verify the validity of the model and correct the error caused in the manufacturing procedure. Besides, the random coefficient regression method reference [19] is adopted to revise our prediction model by taking the uncertainty of test samples caused by material or processing factors into consideration.

## 2. Model Construction of PTH in Multilayered PCB

In the scope of material science failure is defined as performance degradation under continuous stress conditions, and one of the most important failures of PTH is thermal mechanical failure caused by fatigue crack based on the fatigue mechanism. As was mentioned previously, the PTH’s structure is simplified as a one-dimensional rod in most stress-strain models; the influences of the multilayered structure as well as the external and internal pads are ignored because of the difficulty of an analytical solution. This paper proposes an improved model to reveal the constitutive relationship of every point on the PTH in multilayered PCB by combining mechanical equations under the boundary conditions with finite element simulation of the PTH weakness. Subsequently a revised Coffin-Manson model is employed for fatigue life prediction based on the improved multilayered PTH model.

### 2.1. Main Assumption

**Figure 1:**
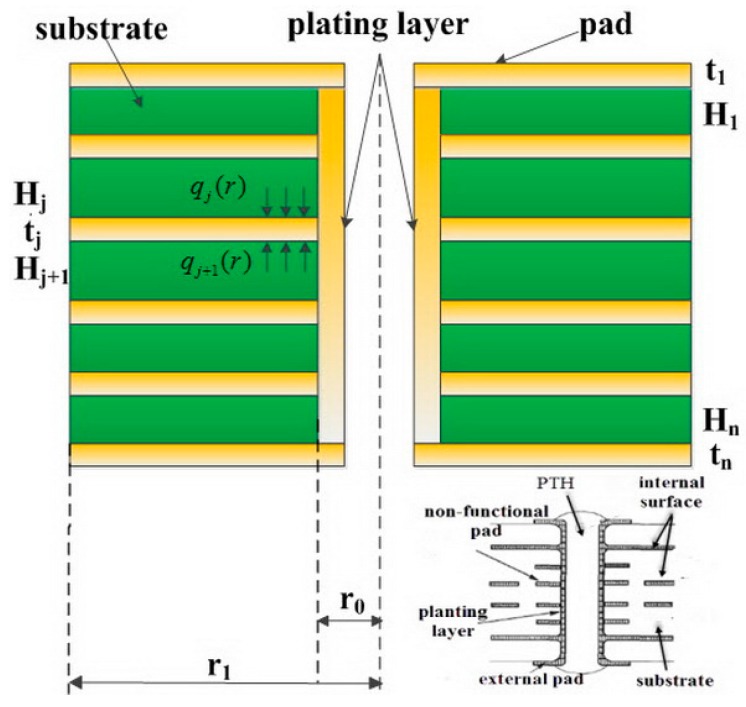
Simplified PTH (plated through hole) structure in multilayered PCB (printed circuit board).

Typical PTH structure includes barrel wall and external pads. Some multilayered PCBs have non-functional pads internally. However, in this paper the internal surface and non-functional pad are simplified as shown in Figure 1. Other assumptions that need to be made are:

(a) The material of the pad and resin is linear elastic.
(b) The shape of the PCB part that affects PTH deformation is a hollow cylinder, with internal diameter equal to the hole-diameter of PTH, and external diameter equal to the pad diameter.
(c) The material of the substrate layer is FR4 epoxy fiberglass cloth, the thicknesses of the layers are the same, barrel layer and pad are all made of copper, and each barrel layer thickness is equal, and so is the pad radius. The substrate and barrel layer materials do not have creep deformation during loading. The plated hole penetrates the whole board, a blind or buried hole is not in the scope of this paper.
(d) The pad is assumed to be a ring-shaped circle plate. On the *j*th layer of the PTH pad two uniform pressure loadings $q_{j}$ and $q_{j+1}$ are applied. The internal edge of the pad is assumed to be simply a supported beam, and the external edge is free.

In this paper, this simplified structure is applied to the constitutive relation of PTH in multilayered PCB, therefore, this model can be named MBPTH (multilayered boards plated through hole) model.

### 2.2. Constitutive Relative of MBPTH

The MBPTH model is established on the basis of beam structure in the Mirman model, with the pressure function of distributed load given as $q_{j}=q_{j}(r)$, $r_{0}<r<r_{1}$, j=1,⋯,n, while the differential equation for deflection function in axisymmetric bending is

(1) $$\frac{d}{dr}\left\{r\frac{d}{dr}\left[\frac{1}{r}\frac{d}{dr}\left(r\frac{d}{dr}w_{j}\right)\right]\right\}=\frac{r}{D_{j}}(q_{j}-q_{j+1}),$$

where $D_{j}$ means the flexural rigidity of the plate, $q_{j}(r)$ means the pressure function of the distributed load at the *j*th layer of PTH, and $\omega_{j}(r)$ means the pad deflection at the *j*th layer of PTH.

The deflection function of MBPTH of the *j*th layer pad can be deduced as

(2) $$w_{j}=A\ln r+Br^{2}\ln r+Cr^{2}+P+w^{*}$$

on account of the uniform loadings $q_{j-1}$ and $q_{j}$, and the particular solution is $w^{*}=\frac{(q_{j}-q_{j+1})r^{4}}{64D_{j}}$. The detailed derivation process is shown in Appendix A.

In addition, there are several boundary conditions that need to be satisfied in Equation (1).

(a) $(M_{r}{)}_{r=r_{0}}=0$, $(M_{r}{)}_{r=r_{1}}=0$, which means the bending moment of the pad at $r=r_{0}$ and $r=r_{1}$ is zero.
  (3) $$\{\begin{array}{l} \frac{d^{2}}{dr^{2}}w_{j}(r_{1})+\frac{\mu }{r_{1}}\frac{d}{dr}w_{j}(r_{1})=0\\ \frac{d^{2}}{dr^{2}}w_{j}(r_{0})+\frac{\mu }{r_{0}}\frac{d}{dr}w_{j}(r_{0})=0 \end{array}$$
  where μ means the Poisson’s ratio of the pad material.
(b) ${\left(\omega \right)}_{r=r_{0}}=0$, which means the deflection of the pad at $r=r_{0}$ is zero
  (4) $$w_{j}(r_{0})=0$$
(c) $(Q_{r}{)}_{r=r_{1}}=0$, which means the sheer stress of the pad at $r=r_{1}$ is zero
  (5) $$r_{1}^{2}\frac{d^{3}}{dr^{3}}w_{j}(r_{1})+r_{1}\frac{d^{2}}{dr^{2}}w_{j}(r_{1})-w_{j}(r_{1})=0$$

All-order derivatives of $w_{j}$ obtained by Equation (2) are then substituted into Equations (3)–(5) to get a system of linear equations, which is related to A/B/C/P. By solving the system of linear equations the four coefficients can be obtained as:

$$A=-\frac{1}{(1-\mu )\left(\frac{1}{r_{0}^{2}-r_{1}^{2}}\right)}\left[-\frac{\left(3+\mu )(q_{j}-q_{j+1})(r_{0}^{2}-r_{1}^{2}\right)}{16D_{j}}+\frac{(1+\mu )(q_{j}-q_{j+1})r_{1}^{2}\ln\frac{r_{0}}{r_{1}}}{4D_{j}}\right],\;B=-\frac{(q_{j}-q_{j+1})r_{1}^{2}}{8D_{j}},\;C=\frac{1}{2(1+\mu )}\left\{\frac{\left(3+\mu )(q_{j}-q_{j+1})(r_{0}^{2}-2r_{1}^{2}\right)}{16}-\frac{(1+\mu )(q_{j}-q_{j+1})r_{1}^{2}\ln r_{0}}{4}+\frac{1}{\left(1-\frac{r_{0}^{2}}{r_{1}^{2}}\right)}\left[-\frac{\left(3+\mu )(q_{j}-q_{j+1})(r_{0}^{2}-r_{1}^{2}\right)}{16}+\frac{(1+\mu )(q_{j}-q_{j+1})r_{1}^{2}}{4}\ln\frac{r_{0}}{r_{1}}\right]\right\}$$

$P=-\frac{(q_{j}-q_{j+1})r_{0}^{4}}{64D_{j}}-A\ln r_{0}-Br_{0}^{2}\ln r_{0}-Cr_{0}^{2}$, the detailed deduction process is presented in Appendix A.

Additionally, the main assumptions (b) and (c) denote that the relationship between distributed pressure and the total strain within each layer of PTH can be represented as Equation (6) and shows,

(6) $$\{\begin{array}{l} q_{j}=E_{Cu}\left[\Delta (\alpha T)-\frac{w_{j+1}(r)-w_{j}(r)}{H_{j}}-\frac{Q_{j}}{E_{E}\cdot \pi (r_{1}^{2}-r_{0}^{2})\cdot t}\right]\\ q_{1}(r)=0,q_{n+1}(r)=0 \end{array}$$

The $Q_{j}$ denotes the extruding force on the surface of the pad, the value of which equals $q_{j}\cdot 2\pi \cdot r_{0}\cdot t$.

Consequently, the value of stress and deflection of each point in the PTH can be calculated by Equations (2) and (6). All the detailed mathematical content can be viewed in Appendix A.

### 2.3. Weak Spot Analysis of MBPTH

MBPTH weakness is deemed to be the place where damage occurs in the PTH and the place that the maximum stress, which is used in the critical plane theory for fatigue life prediction, is imposed. The maximum stress-strain is shown to exist at the junction (corner and barrel) between the external pad and the plating wall by Fu [20], based on which this paper conducts finite element simulation for thermal stress analyses. However, Fu’s work was concentrated on the PTHs of double layered printed wiring board and did not take the uncertainty into consideration, which is caused by the units’ heterogeneity and originated from the manufacturing process.

**Figure 2:**
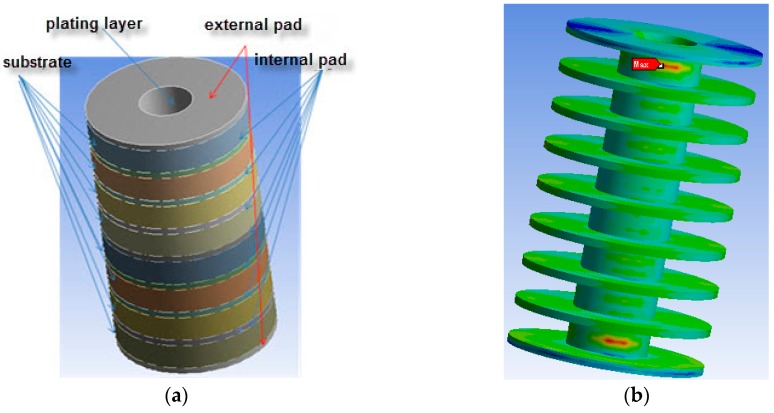
Finite element simulating model of multilayered PTH: (**a**) model constitution and appearance; (**b**) model skeleton with external and internal pads.

With the development of techniques in numerical computation, we are able to locate the weak spot of PTH as well as analyze its stress situation by simulation using finite element analysis software. There are eight substrate layers, seven internal pad layers, two external pad layers, and one planting layer of the MBPTH finite element structure model, as shown in Figure 2a. Material parameters of FEA (finite element analysis) are shown in Table 1.

Setting the thermal load profile by defining the maximum and minimum temperature in ANSYS WORKBENCH, and the distribution of thermal stress contours can be worked out as Figure 2b shows. The result in this paper indicates that the weak spot appears at the junction of the external pad and barrel, which conforms to previous researches. Therefore, the stress situation of the junction is the most significant point to be analyzed.

While at the junction the boundary conditions are $r=r_{0}$, $w_{1}(r_{0})=w_{2}(r_{0})=0$, $q_{1}=0$, hence

(7) $$q_{2}=\frac{E_{Cu}\Delta \alpha T}{1+\frac{E_{Cu}2r_{0}t}{E_{f}({r_{1}}^{2}-{r_{0}}^{2})}}$$

Thus the corresponding coefficients are

$$A=-\frac{1}{(1-\mu )\left(\frac{1}{r_{0}^{2}-r_{1}^{2}}\right)}\left\{\frac{E_{Cu}\Delta \alpha T(3+u)(r_{0}^{2}-r_{1}^{2})}{16D_{1}\left[1+\frac{E_{Cu}2r_{0}t}{E_{E}(r_{1}^{2}-r_{0}^{2})}\right]}-\frac{E_{Cu}\Delta \alpha T(1+\mu )r_{1}^{2}}{4D_{1}\left[1+\frac{E_{Cu}2r_{0}t}{E_{E}(r_{1}^{2}-r_{0}^{2})}\right]}\ln\frac{r_{0}}{r_{1}}\right\},\;B=\frac{E_{Cu}\Delta \alpha Tr_{1}^{2}}{8\left[1+\frac{E_{Cu}2r_{0}t}{E_{E}(r_{1}^{2}-r_{0}^{2})}\right]D_{1}},\;C=\frac{1}{2(1+\mu )}\cdot \frac{E_{Cu}\Delta \alpha T}{1+\frac{E_{Cu}2r_{0}t}{E_{E}(r_{1}^{2}-r_{0}^{2})}}\left\{-\frac{\left(3+\mu )(r_{0}^{2}-2r_{1}^{2}\right)}{16}+\frac{(1+\mu )r_{1}^{2}\ln r_{0}}{4}+\frac{1}{\left(1-r_{0}^{2}/r_{1}^{2}\right)}\left[\frac{\left(3+\mu )(r_{0}^{2}-r_{1}^{2}\right)}{16}-\frac{(1+\mu )r_{1}^{2}}{4}\ln\frac{r_{0}}{r_{1}}\right]\right\}$$

while $D_{j}{|}_{\left(j=1\right)}=D_{1}$ denotes the bending rigidity of the external pad at the first layer, which equals $\frac{E_{Cu}t^{3}}{12(1-\mu^{2})}$, then

(8) $$\{\begin{array}{l} M_{r}(r_{0})=-D_{1}\cdot \left[\frac{d^{2}w(r)}{dr^{2}}+\frac{\mu }{r}\frac{dw(r)}{dr}\right]|r=r_{0}=-D_{1}\cdot \left[\frac{A}{r_{0}^{2}}(\mu -1)+\frac{\mu B\ln r_{0}}{r_{0}}+\frac{B(1+\mu )}{r_{0}}+2C(1+\mu )\right]\\ M_{\theta }(r_{0})=-D_{1}\cdot \left[\frac{1}{r}\frac{dw}{dr}+u\frac{d^{2}w}{dr^{2}}\right]|r=r_{0}=-D_{1}\cdot \left[\frac{A(1-\mu )}{r_{0}^{2}}+\frac{B\ln r_{0}}{r_{0}}+\frac{B(1+\mu )}{r_{0}}+2C(1+\mu )\right] \end{array}$$

and

(9) $$\{\begin{array}{l} \sigma_{r}(r_{0})=\frac{6M_{r}(r_{0})}{t^{2}}=-\frac{E_{Cu}t}{2(1-\mu^{2})}\left[\frac{A}{r_{0}^{2}}(\mu -1)+\frac{\mu B\ln r_{0}}{r_{0}}+\frac{B(1+\mu )}{r_{0}}+2C(1+\mu )\right]\\ \sigma_{\theta }(r_{0})=\frac{6M_{\theta }(r_{0})}{t^{2}}=-\frac{E_{Cu}t}{2(1-\mu^{2})}\left[\frac{A(1-\mu )}{r_{0}^{2}}+\frac{B\ln r_{0}}{r_{0}}+\frac{B(1+\mu )}{r_{0}}+2C(1+\mu )\right]\\ \sigma_{Z}(r_{0})=-q_{2}=\frac{-E_{Cu}\Delta \alpha T}{1+E_{Cu}\cdot 2r_{0}t/\left[E_{E}(r_{1}^{2}-r_{0}^{2})\right]} \end{array}$$

### 2.4. Fatigue Life Prediction Model of MBPTH

The fatigue life of PTH is acknowledged to be dependent on the weak spot, from the perspective of statistics research indicates that the stress and strain of the weakness can reflect the PTH life degradation rule. According to the Von-Mises formula, the $\sigma_{von}$ of MBPTH is

(10) $$\sigma_{von}=\frac{1}{\sqrt{2}}\sqrt{{\left(\sigma_{\gamma }-\sigma_{\theta }\right)}^{2}+{\left(\sigma_{\theta }-\sigma_{z}\right)}^{2}+{\left(\sigma_{z}-\sigma_{\gamma }\right)}^{2}}$$

based on the stress-strain relationship, while the Δε (equivalent strain) of MBPTH is given by

(11) $$\Delta \varepsilon =\left\{ \begin{array}{l} \frac{\sigma_{von}}{E_{Cu}}\;\sigma_{von}<S_{Y}\\ \frac{S_{Y}}{E_{Cu}}+\frac{\sigma_{von}-S_{Y}}{\tilde{E_{Cu}}}\;\sigma_{von}{\text{>}}_{Y} \end{array}\right.$$

where $S_{Y}$ and $\tilde{E_{Cu}}$ denote the yield stress of the pad, and the plastic modulus of the pad, respectively.

Osterman [6] improved the Coffin-Manson model by considering the residual equivalent stress, based on which the equivalent stress and strain values are incorporated into the Coffin-Manson model to evaluate the fatigue life $N_{f}$ of PTH,

(12) $$\frac{\Delta \varepsilon }{2}=\frac{\sigma_{f}-\sigma_{von}}{E_{Cu}}\cdot {\left(2N_{f}\right)}^{b}+\varepsilon_{f}\cdot {\left(2N_{f}\right)}^{c}$$

where $\sigma_{f}$ and $\varepsilon_{f}$ denote the fatigue strength and ductility coefficient of the material respectively, b refers to the fatigue strength exponent (−0.14~−0.06) and c refers to the fatigue ductility exponent (−0.7~−0.5). References show that the coefficients depend on different failure criteria, resistance drifting ΔR/R [9,23]:

(1) 10%, $\sigma_{f}/E_{Cu}=0.0066,b=-0.105,\varepsilon_{f}=0.598,c=-0.6$;
(2) 20%, $\sigma_{f}/E_{Cu}=0.00741,b=-0.11,\varepsilon_{f}=0.709,c=-0.6$;
(3) 50%, $\sigma_{f}/E_{Cu}=0.00763,b=-0.12,\varepsilon_{f}=0.753,c=-0.55$.

## 3. Model Validation

Accuracy of the model can be validated preliminarily by comparing the results of strain/stress analysis from the theoretical model with those from the simulation model in FEA software. Afterwards, thermal cycle tests are conducted to verify the influence of the units’ uncertainty on the fatigue life prediction of the theoretical model.

### 3.1. Comparison between Analytical and Simulation Analysis

As the previous section shows, this paper utilizes the powerful FEA software ANSYS for strain/stress analysis of the MBPTH model. Further, sensitivity analysis is capable of reflecting the influence tendency of geometrical parameters on the pad stress in the MBPTH model, which is also the correct way to reveal the coherence between the theoretical model and the simulation model.

(a) Geometrical parameters of MBPTH used in FEA and the analytical model

**Figure 3:**
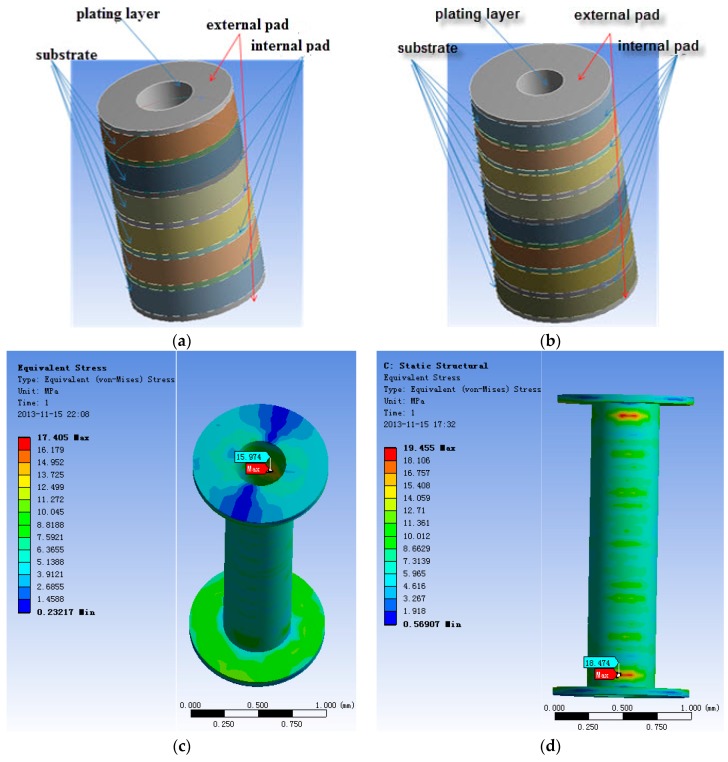
Finite element simulation of multilayered boards plated through hole (MBPTH): 3D-sketch of MBPTH with (**a**) 6 layers and (**b**) 8 layers; (**c**) stress distribution situations of sample (**c**) #3 and (**d**) #8.

According to the IPC standard, the ranges of the different geometrical parameters are obtained: 0.75 mm ≤ *H* (thickness) ≤ 2.5 mm, 0.12 mm ≤ $r_{0}$ (hole radius) ≤ 0.4 mm, 0.24 mm ≤ $r_{1}$ (pad radius) ≤ 0.8 mm, 0.015 mm ≤ *t* (plating thickness) ≤ 0.06 mm. The selected geometry parameters of finite element analysis are shown in Table 2 and the comparison results are shown in Table 3. Figure 3a,b shows the 3D-sketch of MBPTH used for finite element modeling on consideration of a different quantity of layers (six and eight layers). The internal edge of each pad is set to zero degrees of freedom, and the external edge of each pad is free. Based on ten groups geometry data with different size parameters, the thermal load profile condition is from 30 °C to 60 °C with a time slop of 1 °C/min, the dwell times at the maximum and minimum temperature are both 5 min, the ΔT is 30 °C.

Figure 3c,d shows the stress distribution situations of samples #3 and #8 (underlined in Table 3) at one cycle at 30 °C It was confirmed that the maximum equivalent stress exists at the junction between external pad and barrel wall. The simulation computations of $\sigma_{von}$ compared to the theoretical results of $\sigma_{von}$ calculated by axial normal stress ($\sigma_{Z}$), radial normal stress ($\sigma_{r}$) and circumferential normal stress ($\sigma_{\theta }$) are listed in Table 3.

Table 3 also indicates that within an allowable error of magnitude FEA results agree with the results of the MBPTH theoretical model, which partly verifies the accuracy of the model.

(b) Sensitivity analysis of the theoretical model

Single factor approach is applied to analyze the influence tendency of the MBPTH parameters. We try primarily to take the first derivatives of the maximum equivalent stress $\sigma_{von}$ with respect to the geometry parameters in the theoretical model. As a consequence of the theoretical stress data listed in Table 3 in which the values of $\sigma_{r}$ and $\sigma_{\theta }$ are comparatively a small part in the whole stress $\sigma_{von}$, the equivalent stress can be simplified to axial normal stress ($\sigma_{Z}$) for convenience. Besides, the axial normal stress ($\sigma_{Z}$) shown in Equation (9) is in accord with the equivalent stress model which is $\sigma =\frac{(\Delta \alpha T)E_{Cu}}{1+\left[E_{Cu}t/\left(E_{E}\left(r_{1}-r_{0}\right)\right)\right]\cdot \left[2r_{0}/(r_{1}+r_{0})\right]}$ presented by Oien [2].

The first derivatives of $\sigma_{Z}$ with respect to t, $r_{0}$, and $r_{1}$ are obtained as Equation (13) shows,

(13) $$\{\begin{array}{l} \frac{\partial \sigma }{\partial t}=\frac{-2r_{0}{E_{Cu}}^{2}\Delta (\alpha T)}{{\left[E_{f}(r_{1}^{2}-r_{0}^{2})+E_{Cu}\cdot 2r_{0}t\right]}^{2}}<0\\ \frac{\partial \sigma }{\partial r_{0}}=\frac{-2{E_{Cu}}^{2}tE_{f}\Delta (\alpha T)(r_{1}^{2}+r_{0}^{2})}{{\left[E_{f}(r_{1}^{2}-r_{0}^{2})+E_{Cu}\cdot 2r_{0}t\right]}^{2}}<0\\ \frac{\partial \sigma }{\partial r_{1}}=\frac{2{E_{Cu}}^{2}tE_{f}\Delta (\alpha T)r_{1}r_{0}}{{\left[E_{f}(r_{1}^{2}-r_{0}^{2})+E_{Cu}\cdot 2r_{0}t\right]}^{2}}>0 \end{array}$$

as a result, the stress recedes with the increase of t, as well as $r_{0}$, and enhances with the increase of $r_{1}$.

On account that the fatigue life of PTH is negatively related to the equivalent stress, changes in t, $r_{0}$ and $r_{1}$ will definitely affect the life of MBPTH.

**Figure 4:**
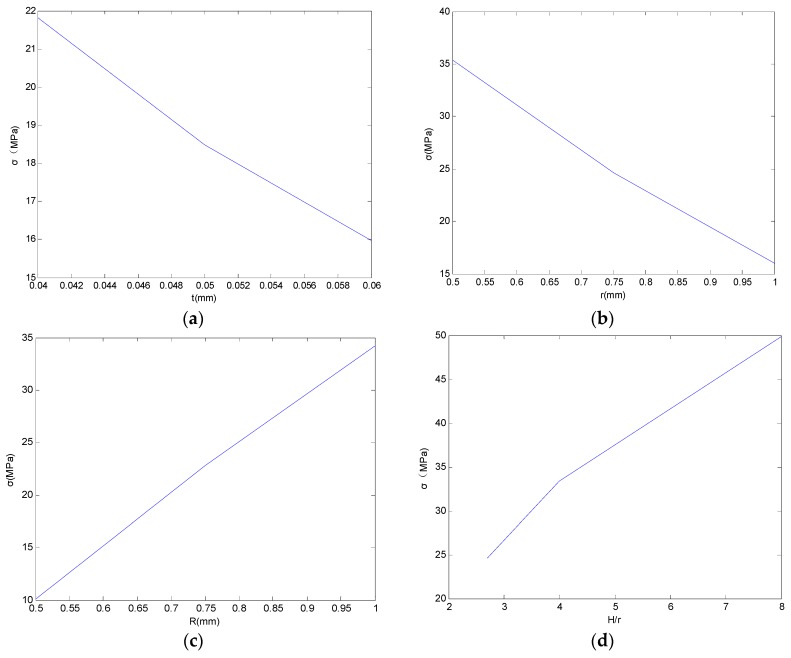
Equivalent stress trends with different geometric parameters: (**a**) plating thickness, t; (**b**) hole radius, $r_{0}$; (**c**) pad radius, $r_{1}$; (**d**) height-diameter ratio, $H/r_{0}$.

Equivalent stress trends can be obtained by processing data in Table 2 and Table 3, as shown in Figure 4, which is consistent with the derivation in Equation (13). Within an allowable error of magnitude, FEA results demonstrate the accuracy of the MBPTH theoretical model, and provide a foundation for estimating the life of MBPTH.

### 3.2. Thermal Cycling Experiment

Failure of PTH may lead to incorrect electronic information on the board level reliability, and the failure assessment of the electronic packaging product is usually based on the criteria of electrical resistance drifting [9]. The thermal cycling test result showed a slowly increasing electrical resistance for every single PTH. Therefore, it was used as a critical breakpoint to get statistical researched data. Up to 5% resistance growth was defined as not having failed. Between 5% and 10% the PTH is preliminary damaged [1]. Thus for the failure criteria of PTH, relative electrical resistance drifting has been widely employed as failure criteria in industry, which differs significantly from 10% to 100% in the open literature [24].

(a) Experimental procedure

Barrel wall fracture occurs largely because of the material processing factors [25], which should be considered in the MBPTH model. Thermal cycling tests can be used to accelerate aging of the PTHs to simulate the few operating years. With the help of these tests it is possible to verify whether the maximum stress areas, which are shown in the simulation results, coincide with the critical failure areas of PTHs. Moreover, the test results can reflect the processing factors to some extent.

**Figure 5:**
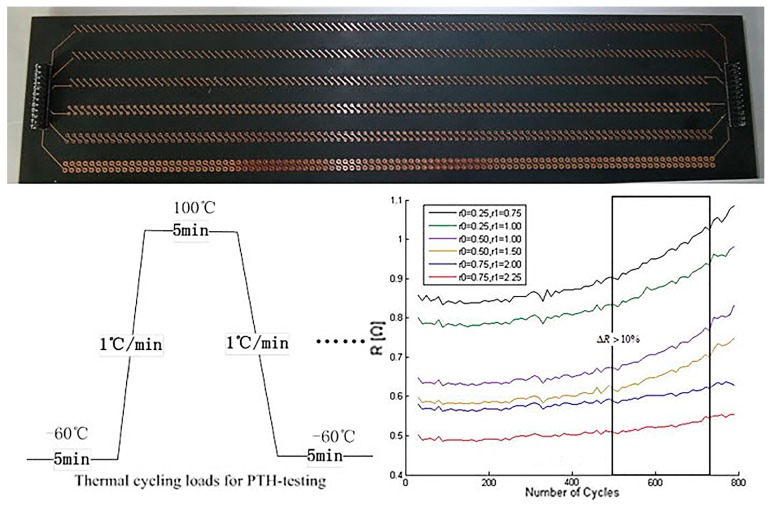
Information of thermal cycling test: specimen, thermal profile, and the number of cycles.

Figure 5 shows the experimental information including test board specimen, thermal cycling loads profile, and the comparison results of electrical resistance measurements.

The PTH test board is designed as a daisy chain structure to ensure better thermal conductivity. The board specimen is divided into six separate daisy chains (0.05 mm plating thickness, six layers, and 1.5 mm substrate thickness). Each chain has two identical paths with 100 vias in series. The main differences between the chains are the radius of the holes ($r_{0}$) and pads ($r_{1}$). The daisy chain makes it possible to measure accurately the sum of the multiple PTHs’ electrical resistance increase, but precise locations of the cracked PTHs are difficult to detect. Therefore, at least up to 10% resistance growth can be defined as a sign that failure occurs in one or more PTHs of the whole chain.

Thermal cycling loads for PTH-testing which are used for this experiment: The temperature cycle was from −60 °C to +100 °C with a time slop of 1 °C/min caused by a one chamber air circulation system. Thermal stress generated by thermal cycling will produce strain in the PTH region, which leads to the increase of electrical resistance of the PTH.

**Figure 6:**
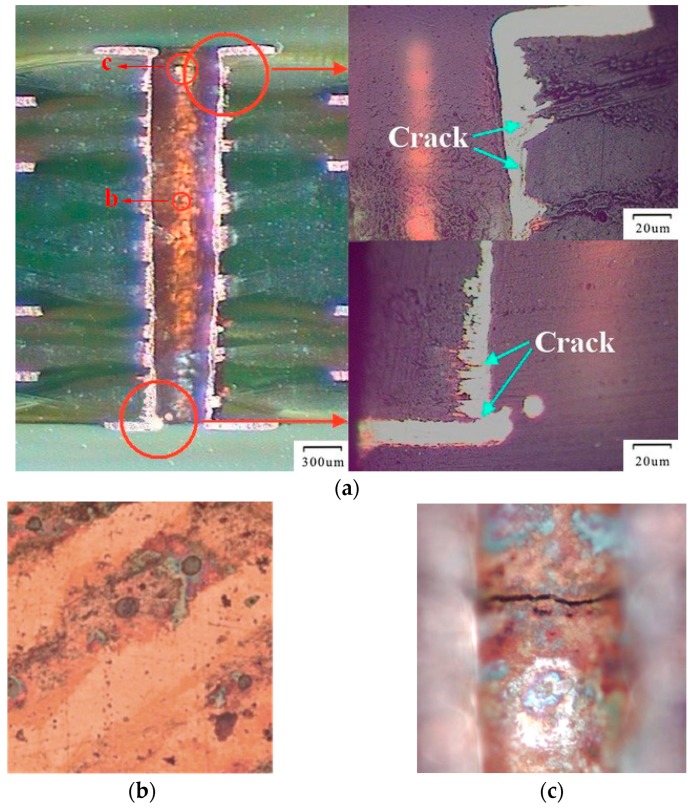
Cracks in the PTH after 800 temperature cycles and different areas of interest: (**a**) the full view and two corners of the PTH; (**b**) surface in the middle of the PTH inner wall; (**c**) surface near the junction (at the top of the PTH inner wall).

The electrical resistance of each PTH daisy chain, with over 800 thermal cycles, is measured to make comparisons. The cycles were applied as the profile requires. It indicated that after 500 thermal cycling steps most PTHs began to show damage and electrical resistance increase over 10%. After the thermal cycle test, scanning electron microscopy (SEM) was used for locating the cracked PTH region, and we observed that numerous corner cracks arose at the weak spot offered by FEA, as shown in Figure 6.

The surface of the sample micro-section presented in Figure 6a seemed very rough, so we kept on polishing the sample to the inner wall of the PTH to find out whether the rough surface affected the occurrence of the cracks. As Figure 6a,b show, even though the degrees of surface rough at the top or in the middle of the PTH inner wall are similar, cracks still existed near the junction of the pad and the wall as we assumed. Therefore, it is reasonable to say that the cycling load is the key reason of initial crack, and maybe the rough surface is due to the procedure of making the sample micro-section.

(b) Random coefficient method for model revision

The uncertainty from material and manufacturing variabilities may have an effect on the performance degradation of products of the same batch, so the random coefficient method is employed to revise our prediction model for the specimen.

In Table 4 the variables of all the PTHs daisy chains are listed, the MBPTH model can be used for computing the theoretical Δε and $N_{f}$.

By transforming experimental $N_{f}$ into corresponding strain Δε by Equation (12), it can be found that the data from experiment and theoretical model conform approximately to the linear rule. Hence we can assume a multiplication coefficient in the modified formula of the prediction model as

(14) $$\{\begin{array}{c} \Delta \varepsilon_{eff}=K\cdot \Delta \varepsilon \\ \frac{\Delta \varepsilon_{eff}}{2}=\frac{\sigma_{f}-\sigma_{von}}{E}{\left(2N_{f}\right)}^{b}+\varepsilon_{f}{\left(2N_{f}\right)}^{c} \end{array}$$

where $\Delta \varepsilon_{eff}$ refers to effective stress. By fitting the data in Table 4 with the least square method, the multiplication coefficient of the equivalent strain can be calculated and the value of *K* approximates 0.978.

## 4. Conclusions

This paper proposed a new multilayered PTH model, which could reasonably show the constitutive relation of multilayered, simulation results of stress-strain distribution, and comparison of the verification test. The developed model can be used to predict the lifetime of PTH with multi-layers.

Reasonable assumptions of structure simplification and material properties are proposed and the analytical results of the maximum stress-strain situation in multilayered PTH are given. The weak spot of multilayered PTH and the influence of geometry parameters on the maximum stress were investigated by FEA. Moreover, their final effects on the PTH lifetime was verified and revised by experimental data. It is worth noting that the physics of the failure model proposed in this paper contributed to a more precise prediction for PTH in practice. While the uncertainty in the model was considered only roughly, more attention should be focused on the uncertainty of the material and the manufacturing process for specific PTH objects in future research.

## Figures and Tables

**Table 1:** Material parameters used in finite element analysis (FEA) and the analytical model [21,22].

Material		Parameter								
		Elastic Modulus (Gpa)	CTE ppm/°C	Poissn Ratio	Shear Modulus (Gpa)	Density (kg/m^3^)	Specific Heat J/(kg·°C)	Yield Strength (Mpa)		Yield Limit (Mpa)
								Tensile	Compressive	
Barrel/Pad (Copper)		130	17	0.35	48	8300	390	280	280	430
Substrate (FR4)	C	17.2	18.2	0.13	5.07	1500	1000	350	350	550
	L	7.45	58.7	0.42	2.17					

The C in Table 1 refers to crosswise while the L refers to lengthways. CTE = coefficient of thermal expansion.

**Table 2:** Geometry parameters setting in simulation and theoretical.

No.	$r_{0}$ (mm)	$r_{1}$ (mm)	*t* (mm)	Layers	*H* (mm)
1	0.125	0.25	0.06	6	2
2	0.125	0.375	0.06	6	2
3	0.25	0.5	0.06	6	2
4	0.25	0.75	0.06	6	2
5	0.375	0.75	0.06	6	2
6	0.125	0.25	0.05	8	2
7	0.125	0.375	0.05	8	2
8	0.25	0.5	0.05	8	2
9	0.25	0.75	0.05	8	2
10	0.375	0.75	0.05	8	2

**Table 3:** Comparison results $\sigma_{von}$ of simulation and theoretical model.

No.	Simulation $\sigma_{von}$ (MPa)	Theoretical (MPa)				Error
		$\sigma_{von}$	$\sigma_{Z}$	$\sigma_{\gamma }$	$\sigma_{\theta }$	
1	8.8739	8.913	9.154	0.0457	0.45	4.41‰
2	19.717	19.930	20.477	0.113	1.01	10.84‰
3	15.974	15.977	16.415	0.0825	0.818	0.22‰
4	31.257	31.699	32.561	0.163	1.61	14.15‰
5	21.706	21.754	22.317	0.115	1.04	2.22‰
6	10.297	10.455	10.737	0.0537	0.526	15.36‰
7	22.86	22.751	23.369	0.1168	1.154	−4.74‰
8	18.474	18.415	18.916	0.0956	0.935	−3.19‰
9	35.393	35.167	36.113	0.1868	1.7576	−6.38‰
10	24.636	24.703	25.356	0.1268	1.214	2.74‰

The two underlined simulation equivalent stresses are the results of Figure 3c,d.

**Table 4:** Comparison of theoretical and experimental results for selected geometry parameters.

No.	$r_{0}$ (mm)	$r_{1}$ (mm)	Theoretical Δε	Theoretical $N_{f}$	Experimental Δε	Experimental $N_{f}$
1	0.25	0.75	0.04212	470	0.04047	494
2	0.25	1	0.03845	495	0.03289	512
3	0.5	1	0.02930	565	0.02591	587
4	0.5	1.5	0.02651	571	0.02380	595
5	0.75	2	0.01972	729	0.01513	755
6	0.75	2.25	0.01601	760	0.01440	794

## Nomenclature

r	Extent along the PTH radial axes
$r_{0}$	Radius of hole
$r_{1}$	Radius of Pad
$\sigma_{r}$	Radial normal stress
$\sigma_{\theta }$	Circumferential normal stress
$\sigma_{Z}$	Axial normal stress
$\sigma_{von}$	Equivalent stress
Δε	Equivalent strain
μ	Poisson’s ratio
t	Plating thickness
$D_{j}$	Flexural rigidity of plate
ΔT	Temperature difference
α	Coefficient of thermal expansion of material
Δ(αT)	Thermal strain, which value equals $(\alpha_{substrate}-\alpha_{barrel})\cdot \Delta T$
$E_{Cu}$	Elastic modulus of barrel material
$E_{E}$	Elastic modulus of substrate material
$N_{f}$	Number of thermo-mechanical failure cycle
$q_{j}(r)$	Pressure function of distributed load at the *j*th layer of PTH
$\omega_{j}(r)$	Pad deflection at the *j*th layer of PTH

## Appendix A. Mathematical Deduction of MBPTH Constitutive Relation

The MBPTH model is established on the basis of beam structure in the Mirman model, with the pressure function of distributed load given as $q_{j}=q_{j}(r)$, $r_{0}<r<r_{1}$, j=1,⋯,n, while the differential equation for deflection function in axisymmetric bending is as Equation (1) shows

(1) $$\frac{d}{dr}\left\{r\frac{d}{dr}\left[\frac{1}{r}\frac{d}{dr}\left(r\frac{d}{dr}w_{j}\right)\right]\right\}=\frac{r}{D_{j}}(q_{j}-q_{j+1})$$

where $D_{j}$ means the flexural rigidity of plate, $q_{j}(r)$ means pressure function of distributed load at the *j*th layer of PTH, and $\omega_{j}(r)$ means the pad deflection at the *j*th layer of PTH. Equation (1) can be used to derive deflection function of MBPTH, and the derivation process is shown in Equations (A1)–(A3).

Equation (A1) is the expanded expression of Equation (1).

(A1) $$\frac{d^{4}w_{j}}{dr^{4}}+\frac{2}{r}\frac{d^{3}w_{j}}{dr^{3}}-\frac{1}{r^{2}}\frac{d^{2}w_{j}}{dr^{2}}+\frac{1}{r^{3}}\frac{dw_{j}}{dr}=\frac{q_{j}-q_{j+1}}{D_{j}}$$

Let the $r=e^{x}$, thus x=lnr, and we have

(A2) $$\frac{d^{4}w}{dx^{4}}-4\frac{d^{3}w}{dx^{3}}+4\frac{d^{2}w}{dx^{2}}=\frac{q_{j}-q_{j+1}}{D_{j}}$$

The characteristic equation of Equation (3) is $\lambda^{4}-4\lambda^{3}+4\lambda^{2}=0$, of which the general equation is $w=At+Bte^{2t}+Ce^{2t}+P$. As a result, the deflection function of the *j*th layer pad is

(A3) $$w_{j}=A\ln r+Br^{2}\ln r+Cr^{2}+P+w^{*}$$

on account of the uniform loadings $q_{j-1}$ and $q_{j}$, and the particular solution is $w^{*}=\frac{(q_{j}-q_{j+1})r^{4}}{64D_{j}}$.

With the several boundary conditions: (1) $(M_{r}{)}_{r=r_{0}}=0$, $(M_{r}{)}_{r=r_{1}}=0$; (2) ${\left(\omega \right)}_{r=r_{0}}=0$; (3) $(Q_{r}{)}_{r=r_{1}}=0$, as shown in Equation (A4),

(A4) $$\{\begin{array}{l} \frac{d^{2}}{dr^{2}}w_{j}(r_{1})+\frac{\mu }{r_{1}}\frac{d}{dr}w_{j}(r_{1})=0\\ \frac{d^{2}}{dr^{2}}w_{j}(r_{0})+\frac{\mu }{r_{0}}\frac{d}{dr}w_{j}(r_{0})=0\\ w_{j}(r_{0})=0\\ r_{1}^{2}\frac{d^{3}}{dr^{3}}w_{j}(r_{1})+r_{1}\frac{d^{2}}{dr^{2}}w_{j}(r_{1})-w_{j}(r_{1})=0 \end{array}$$

All-order derivatives of $w_{j}$ obtained by Equation (4) are then substituted into Equations (5)–(7) to get a system of linear equations, which is related to A/B/C/P, and these coefficients can be solved as $A=\frac{\Delta_{1}}{\Delta_{0}}$, $B=\frac{\Delta_{2}}{\Delta_{0}}$, $C=\frac{\Delta_{3}}{\Delta_{0}}$, $P=\frac{\Delta_{4}}{\Delta_{0}}$, in which

$$\Delta_{0}=|\begin{array}{cccc} a_{1} & a_{2} & a_{3} & a_{4}\\ b_{1} & b_{2} & b_{3} & b_{4}\\ c_{1} & c_{2} & c_{3} & c_{4}\\ d_{1} & d_{2} & d_{3} & d_{4} \end{array}|,\;\Delta_{1}=|\begin{array}{cccc} a_{0} & a_{2} & a_{3} & a_{4}\\ b_{0} & b_{2} & b_{3} & b_{4}\\ c_{0} & c_{2} & c_{3} & c_{4}\\ d_{0} & d_{2} & d_{3} & d_{4} \end{array}|,\;\Delta_{2}=|\begin{array}{cccc} a_{1} & a_{0} & a_{3} & a_{4}\\ b_{1} & b_{0} & b_{3} & b_{4}\\ c_{1} & c_{0} & c_{3} & c_{4}\\ d_{1} & d_{0} & d_{3} & d_{4} \end{array}|,\;\Delta_{3}=|\begin{array}{cccc} a_{1} & a_{2} & a_{0} & a_{4}\\ b_{1} & b_{2} & b_{0} & b_{4}\\ c_{1} & c_{2} & c_{0} & c_{4}\\ d_{1} & d_{2} & d_{0} & d_{4} \end{array}|,\;\Delta_{4}=|\begin{array}{cccc} a_{1} & a_{2} & a_{3} & a_{0}\\ b_{1} & b_{2} & b_{3} & b_{0}\\ c_{1} & c_{2} & c_{3} & c_{0}\\ d_{1} & d_{2} & d_{3} & d_{0} \end{array}|,$$

where $a_{0}=\frac{(q_{j}-q_{j+1})r_{0}^{4}}{64D_{j}}$, $a_{1}=\ln r_{0}$, $a_{2}=r_{0}^{2}\ln r_{0}$, $a_{3}=r_{0}^{2}$, $a_{4}=1$;

$$b_{0}=\frac{\left(q_{j}-q_{j+1})(3r_{0}^{2}+ur_{0}^{2}\right)}{16D_{j}},\;b_{1}=\frac{1}{r_{0}^{2}}(u-1),\;b_{2}=(2\ln r_{0}+3+2u\ln r_{0}+u),\;b_{3}=(2+2u),\;b_{4}=0;$$

$$c_{0}=\frac{\left(q_{j}-q_{j+1})(3r_{1}^{2}+ur_{1}^{2}\right)}{16D_{j}},\;c_{1}=\frac{1}{{r_{1}}^{2}}(u-1),\;c_{2}=(2\ln r_{1}+3+2u\ln r_{1}+u),\;c_{3}=(2+2u),\;c_{4}=0;$$

$$d_{0}=\frac{\left(q_{j}-q_{j+1})(12{r_{1}}^{3}-r_{1}^{4}\right)}{64D_{j}},\;d_{1}=-\frac{3}{r_{1}}-\ln r_{1},\;d_{2}=(2r_{1}\ln r_{1}+5r_{1})-r_{1}^{2}\ln r_{1},\;d_{3}=2r_{1}-r_{1}^{2},\;d_{4}=-1;$$

By solving the system of linear equations the four coefficients can be obtained as:

$$A=-\frac{1}{(1-\mu )\left(\frac{1}{r_{0}^{2}-r_{1}^{2}}\right)}\left[-\frac{\left(3+\mu )(q_{j}-q_{j+1})(r_{0}^{2}-r_{1}^{2}\right)}{16D_{j}}+\frac{(1+\mu )(q_{j}-q_{j+1})r_{1}^{2}\ln\frac{r_{0}}{r_{1}}}{4D_{j}}\right],\;B=-\frac{(q_{j}-q_{j+1})r_{1}^{2}}{8D_{j}},\;C=\frac{1}{2(1+\mu )}\left\{\frac{\left(3+\mu )(q_{j}-q_{j+1})(r_{0}^{2}-2r_{1}^{2}\right)}{16}-\frac{(1+\mu )(q_{j}-q_{j+1})r_{1}^{2}\ln r_{0}}{4}+\frac{1}{\left(1-\frac{r_{0}^{2}}{r_{1}^{2}}\right)}\left[-\frac{\left(3+\mu )(q_{j}-q_{j+1})(r_{0}^{2}-r_{1}^{2}\right)}{16}+\frac{(1+\mu )(q_{j}-q_{j+1})r_{1}^{2}}{4}\ln\frac{r_{0}}{r_{1}}\right]\right\},\;P=-\frac{(q_{j}-q_{j+1})r_{0}^{4}}{64D_{j}}-A\ln r_{0}-Br_{0}^{2}\ln r_{0}-Cr_{0}^{2}.$$

Additionally, from the assumptions (b) and (c) the relationship between distributed pressure and the total strain within each layer of PTH can be represented as Equation (6) shows,

(6) $$\{\begin{array}{l} q_{j}=E_{Cu}\left[\Delta (\alpha T)-\frac{w_{j+1}(r)-w_{j}(r)}{H_{j}}-\frac{Q_{j}}{E_{E}\cdot \pi (r_{1}^{2}-r_{0}^{2})\cdot t}\right],\;\mathrm{for}\;j=2,\ldots n\\ q_{1}(r)=0,q_{n+1}(r)=0 \end{array}$$

The $Q_{j}$ denotes extruding force on the surface of pad, the value of which equals $q_{j}\cdot 2\pi \cdot r_{0}\cdot t$.

Consequently, the value of stress and deflection of each point in the PTH can be calculated.

## References

[1] Nowak T., Schacht R., Walter H. Experimental and numerical reinvestigation for lifetime-estimation of plated through holes in printed circuit boards. Proceedings of the 2011 17th International Workshop on Thermal Investigations of ICs and Systems (THERMINIC).

[2] Oien M. A simple model for the thermo-mechanical deformations of plated-through-holes in multilayered printed wiring boards. Proceedings of the 14th International Reliability Physics Symposium.

[3] Engelmaier W. (1987). Results of the IPC copper foil ductility round-robin study. Testing of Metallic and Inorganic Coatings (STP947).

[4] Xie J., Kang R., Zhang Y. A PTH reliability model considering barrel stress distributions and multiple PTHs in a PWB. Proceedings of the 44th Annual IEEE International Reliability Physics Symposium.

[5] Mirman B. (1988). Mathematical model of a plated-through hole under a load induced by thermal mismatch. IEEE Trans. Compon. Hybrids Manuf. Technol..

[6] Osterman M. (2002). CALCE Plated through Hole Fatigue Model.

[7] Iannuzzelli R. Predicting plated-through-hole reliability in high temperature manufacturing processes. Proceedings of the 41st Electronic Components and Technology Conference.

[8] Wu J.-H., Meng-Chieh L., Tzeng-Cherng L. Comparison the reliability of small plated-through hole with different diameters under thermal stress. Proceedings of the 2011 6th International Microsystems, Packaging, Assembly and Circuits Technology Conference (IMPACT).

[9] Su F., Mao R., Xiong J. (2012). On thermo-mechanical reliability of plated-through-hole (PTH). Microelectron. Reliab..

[10] Peng W., Huang H.-Z., Xie M. (2013). A Bayesian approach for system reliability analysis with multilevel pass-fail, lifetime and degradation data sets. IEEE Trans. Reliab..

[11] Xiao N.-C., Li Y.-F., Wang Z. (2013). Bayesian Reliability Estimation for Deteriorating Systems with Limited Samples Using the Maximum Entropy Approach. Entropy.

[12] Chiachío M., Chiachío J., Rus G., Beck J.L. (2014). Predicting fatigue damage in composites: A Bayesian framework. Struct. Saf..

[13] Chiachío J., Chiachío M., Saxena A., Sankararaman S., Rus G., Goebel K. (2015). Bayesian model selection and parameter estimation for fatigue damage progression models in composites. Int. J. Fatigue.

[14] Corbetta M., Sbarufatti C., Manes A., Giglio M. (2014). Continuous Crack Growth Monitoring and Residual Life Prediction under Variable-amplitude Loading Conditions. Procedia Eng..

[15] Knadle K.T., Jadhav V.R. Proof is in the PTH—Assuring via reliability from chip carriers to thick printed wiring boards. Proceedings of the 55th Electronic Components and Technology Conference.

[16] Sun B., Pan W., Wang Z., Yung W.K.C. (2015). Envelope probability and EFAST-based sensitivity analysis method for electronic prognostic uncertainty quantification. Microelectron. Reliab..

[17] Pan R., Xu X., Juarez J. (2016). Bayesian analysis of low-cycle fatigue failure in printed wiring boards. Case Stud. Eng. Fail. Anal..

[18] Chen T.C., Tsai Y.L., Hsu C.F., Dow W.-P., Hashimoto Y. (2016). Effects of Brighteners in a Copper Plating Bath on Throwing Power and Thermal Reliability of Plated through Holes. Electrochim. Acta.

[19] Zhang Z., Si X., Hu C., Kong X. (2015). Degradation modeling-based remaining useful life estimation: A review on approaches for systems with heterogeneity. Proc. Inst. Mech. Eng. Part O J. Risk Reliab..

[20] Fu C., Ume I.-C., Mcdowell D.-L. (1998). Thermal stress and fatigue analysis of plated-through holes using an internal state variable constitutive model. Finite Elem. Anal. Des..

[21] Li Y.-Q., Liu P., Feng L.-Y. (2010). Research on the PCB barrel crack. Electroplat. Finish..

[22] Bhandarkar S., Dasgupta A., Pecht M. Effects of Voids in Solder-Filled Plated-Through Holes. Proceedings of the 33rd IPC Annual Meeting.

[23] Gu M. (2001). China Aeronautical Materials Handbook.

[24] Pecht M., Dasgupta A., Naqvi S. (1991). Transient thermal stress analysis of a plated through hole subjected to wave soldering. J. Electron. Packag..

[25] (1988). Round Robin Reliability Evaluation of Small Diameter Plated through Holes in Printed Wiring Boards.

